# Dengue rhabdomyolysis successfully treated with hemoperfusion using CytoSorb® in combination with continuous renal replacement therapy: a case report

**DOI:** 10.1186/s13256-024-04661-6

**Published:** 2024-07-19

**Authors:** Piyum Samarasingha, Harindra Karunatilake, Ananda Jayanaga, Hansani Jayawardhana, Dilshan Priyankara

**Affiliations:** National Hospital of Sri Lanka, Colombo, Sri Lanka

**Keywords:** Dengue rhabdomyolysis, Acute kidney injury, CytoSorb, Continuous renal replacement therapy

## Abstract

**Background:**

Dengue fever is a mosquito-borne viral infection with a broad spectrum of clinical manifestations. Expanded dengue syndrome includes unusual manifestations that do not fall into the categories of dengue fever, dengue hemorrhagic fever, or dengue shock syndrome. Rhabdomyolysis causing acute renal failure in dengue is one such unusual manifestation, the pathophysiology of which is incompletely understood.

**Case presentation:**

We describe a 21-year-old Sri Lankan man with dengue fever who developed severe rhabdomyolysis and acute kidney injury with extremely high creatinine phosphokinase levels (> 2 million U/L). Management of this patient was challenging as his creatinine phosphokinase kept rising with persistent anuria despite hydration, intermittent hemodialysis, and, later, continuous venovenous hemodiafiltration. Further therapeutic options were explored, and CytoSorb® adsorber was added as an adjunct to continuous venovenous hemodiafiltration, following which we observed a marked reduction in his creatinine phosphokinase and myoglobin levels over the next 12 hours and complete renal recovery over the next 5 weeks.

**Conclusion:**

We report a rare case of significant rhabdomyolysis secondary to dengue infection leading to acute kidney injury. Continuous venovenous hemodiafiltration performed with the hemofilter Pecopen 140 was ineffective, and the addition of CytoSorb® adsorber as an adjunct therapy to continuous venovenous hemodiafiltration may have a potential benefit in removing high-molecular-weight proteins such as myoglobin.

## Background

Dengue fever is a mosquito-borne viral infection with a rising global incidence of more than 50-fold over the past decade with geographical expansion. It poses a significant health and economic burden, and the majority (70%) of the at-risk population belongs to Southeast Asia and Western Pacific regions [1].

Dengue has a wide spectrum of clinical presentations ranging from mild febrile illness to severe illness characterized by capillary leakage with or without hemorrhage and dengue shock syndrome. However, dengue may present with certain atypical features that fall outside the above categories. Such presentations have been described under expanded dengue syndrome and include a spectrum of multisystemic disease manifestations [1–3]. Rhabdomyolysis has been described in several case reports as one such atypical manifestation, which has frequently led to acute renal failure.

We present a case of expanded dengue syndrome, with rhabdomyolysis and acute renal failure, with extremely high levels of CPK, which was successfully managed with hemoperfusion using CytoSorb® in combination with continuous renal replacement therapy (CRRT).

## Case report

A 21-year-old previously healthy Sri Lankan man weighing 55 kg presented with a 2-day history of fever and severe myalgia. On admission, he was febrile and had stable vital parameters. His initial full blood count showed a normal platelet count of 292,000/μL. His dengue nonstructural protein 1 (NS1) antigen was positive. He was managed as having dengue fever symptomatically with adequate oral hydration. On day 2 of the hospital stay, he complained of reduced urine output and passage of dark urine. On examination, he was febrile and tachycardic with normal blood pressure and had severe muscle tenderness. There was no clinical or ultrasonographic evidence of fluid extravasation to suggest dengue hemorrhagic fever. He continued to be anuric for the next few hours, and his creatinine level was elevated (1.5 mg/dL) with a creatine phosphokinase (CPK) level of 314,221 U/L (normal range 25–174 U/L). A diagnosis of rhabdomyolysis (RM) and acute kidney injury (AKI) was made. He also had raised serum lactate dehydrogenase (LDH) of 23,507 U/L, aspartate transaminase (AST)of 4700 U/L, and phosphate of 7.4 mg/dL with low ionized calcium of 0.99 mmol/L, which was consistent with rhabdomyolysis. However, serum potassium remained normal. He was managed with hydration with intravenous 0.9% saline 2 ml/kg/hour. Despite adequate hydration, he remained anuric, with rising creatinine up to 2.4 mg/dL.

**Fig. 1:**
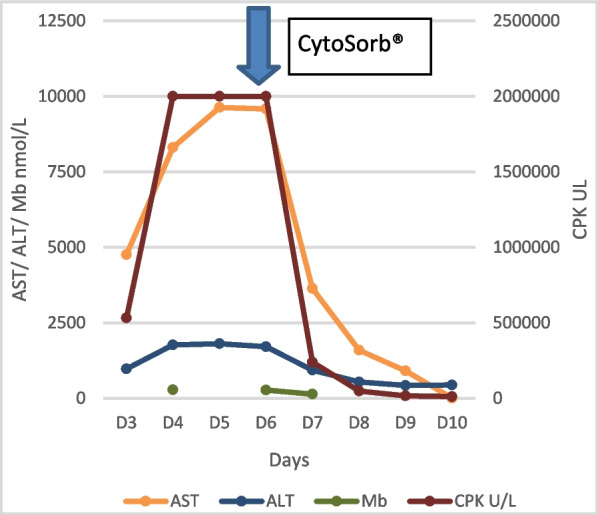
Trends in biochemistry. Progression of aspartate transaminase, alanine transaminase, myoglobin, and creatine phosphokinase during intensive care unit stay. A rapid reduction of creatine phosphokinase, aspartate transaminase, and myoglobin was noted after commencement of CytoSorb® absorber as an adjunct. The arrow indicates the time of commencement of CytoSorb® absorber as an adjunct

Due to ongoing AKI and rhabdomyolysis, he was transferred to the intensive care unit. He was initiated on continuous venovenous hemodiafiltration (CVVHDF, Pecopen 140 filter) with a blood flow rate of 150–200 mL/min and a dose of 25–30 mL/kg/hour (50% filtration, 50% dialysis) with fluid removal of 80–100 cc/hour. He remained hemodynamically stable without any inopressor support. However, despite this, the patient remained anuric, and within the next 48 hours, his CPK level steadily rose to more than 2 million U/L and his myoglobin level was 276 nmol/L (normal range < 80 nmol/L). Further therapeutic options were explored, and CytoSorb® adsorber was added as an adjunct therapy to CVVHDF. At 12 hours into the initiation of CytoSorb®, the CPK dropped to 807,700 U/L, and at 24 hours his CPK value dropped to 240,497 U/L. The myoglobin level dropped from 276 nmol/L to 139.2 nmol/L at 24 hours after initiating CytoSorb® (Fig. 1). CytoSorb® was continued for 36 hours, and continuous renal replacement therapy (CRRT) was continued for the next 72 hours. His CPK and myoglobin levels continued to decline and dropped to 16,598 U/L and 98 nmol/L, respectively. However, his renal recovery lagged and remained oliguric for the next 2 weeks and required further episodes of intermittent hemodialysis. Table 1 below summarizes the investigation results of the patient during the course of the hospital stay.

**Table 1:** Investigations of the patient

	Day 3	Day 4	Day 5	Day 6	Day 7	Day 8	Day 9	Day 10
WCC 10^9^/L	10.6	6.2	4	4.8	6.52	6.77	9.53	9.72
HB (g/dL)	15.2	13.6	13.9	14.3	12.9	11.7	12.1	11.1
Plt × 10^9^/L	106	77	30	37	73	92	171	226
Cr (mg/dl)	2.9	2.99	4.6	4.76	3	3.2	4.03	5.73
K (mmol/l)	4.2	4.5	5.1	5.2	3.6	4	3.7	4.2
AST (U/L)	4757	8307	9633	9584	3640	1599	910	13
ALT (U/L)	978	1773	1810	1712	932	547	430	443
CPK (U/L)	532,788	2,000,000	2,000,000	2,000,000	240,497	48,881	16,598	12,018
Mb (nmol/L)		287		276	139.2		98	
CRP (mg/dL)	39.9	33.7	34.8				34.2	

*WCC* white cell count, *HB* hemoglobin, *Plt* platelets, *Cr* serum creatinine, *K* potassium, *AST* aspartate transaminase, *ALT* alanine transaminase, *CPK* creatine phosphokinase, *Mb* myoglobin, *CRP* C-reactive protein

A renal biopsy was done on day 13 of his hospital stay. It showed acute tubular injury with myoglobin, red cells, and granular casts, which were compatible with rhabdomyolysis-induced AKI.

Electromyogram (EMG) findings were compatible with resolving myositis. A muscle biopsy was done on day 20, which showed moderate endomysial inflammation with lymphocytes and plasma cells and individual fiber atrophy with fat infiltration. Focal areas of myocyte necrosis and regeneration were also noted with mild perivascular lymphocytic infiltration. These findings were compatible with polymyositis. The extractable nuclear antigen antibody panel was negative.

He did not have a family history of metabolic myopathies, and he did not give an account of any previous episodes of muscle pain or weakness following exertion. His presentation was acute, and he denied any long-standing proximal muscle weakness. He was clinically and biochemically [free thyroxine (fT4) 1.1 ng/dL, thyroid-stimulating hormone (TSH) 3.012 mIU/L] euthyroid. He was not on any drugs known to cause rhabdomyolysis. We also screened him for other infections known to cause myositis. His leptospirosis antibody, human immunodeficiency virus (HIV) screening, cytomegalovirus (CMV), Epstein–Barr virus (EBV), herpes simplex virus (HSV), and coronavirus disease (COVID) polymerase chain reaction (PCR) were all negative. His dengue infection was confirmed by positive NS1 antigen at the onset of the illness and positive dengue immunoglobulin M (IgM) antibodies after the fifth day of the illness.

At the time of discharge, he was asymptomatic with satisfactory urine output, and his CPK was 434 U/L with a creatinine of 3.0 mg/dL. He was reviewed after 2 weeks; his creatinine and CPK levels had returned to normal range, and he made a complete recovery.

## Discussion

Rhabdomyolysis occurs due to damage to skeletal myocytes, which release toxic intracellular contents into the systemic circulation. This leads to elevated plasma CPK and myoglobinuria and poses a risk of developing AKI. The pathophysiology of rhabdomyolysis-induced AKI is complex. Myoglobin is a protein that can bind to oxygen and is freely filtered at the glomerulus. Filtered myoglobin can precipitate in the tubules of the nephron and can cause acute tubular injury and AKI. Furthermore, oxidative damage causing direct tubular injury, hypovolemia, and renal vasoconstriction contributes to renal injury [4, 5]. Blood levels of myoglobin and CPK are the most sensitive markers of muscle injury, and a CPK value of more than 5000 U/L is said to be associated with the development of AKI [6].

Coxsackie virus, influenza A and B, Epstein–Barr virus, and HIV are recognized viruses that cause rhabdomyolysis [7]. Rhabdomyolysis in dengue has been rarely reported, and the exact pathophysiological mechanism causing dengue rhabdomyolysis still needs to be fully understood [8]. There are assumptions that myotoxic cytokines, such as tumor necrosis factor α (TNFα) and interferon-alpha (IFNα), produced during dengue infection are involved in myositis [9]. In published case reports, CPK value ranged between 5000 and 325,000 U/L. Despite high CPK values, some patients have not developed AKI, and some affected individuals have died due to multiorgan failure.

Treatment of rhabdomyolysis is aimed at enhancing the clearance of myoglobin and managing AKI-related complications such as hyperkalemia and acidosis. Therefore, the primary management principles of rhabdomyolysis include hydration and maintaining a good urinary flow. Moderate to severe rhabdomyolysis may require 150–200 mL/hour crystalloids to maintain euvolemia and higher urine output (2–3 mL/kg/hour). This poses a challenge in dengue hemorrhagic fever as higher rates of fluid resuscitation may aggravate fluid leakage and contribute to fluid overload. However, our patient did not show evidence of fluid leak into the serosal cavities, simplifying fluid management.

Removal of myoglobin may prevent renal damage or may limit the progression and extent of AKI. Myoglobin has a molecular weight of 17.8 kDa (middle-size molecule). Extracorporeal blood purification (EBP) devices support organ failures in critically ill patients. CRRT is one form of EBP technology widely used worldwide to support failing kidneys. High-flux filters (pore size 3–4 nm) in convective modes (continuous venovenous hemofiltration—CVVH, CVVHDF) are capable of removing myoglobin compared with diffusive methods such as continuous venovenous haemodialysis (CVVHD) [10]. Furthermore, it was shown that the clearance of middle-size molecules such as β2 microglobulin and myoglobin is better with hemodialysis with middle cutoff (MCO) dialyzer than with hemodiafiltration with the high-flux dialyzer [11]. Therefore, MCO dialyzers can clear myoglobin during CRRT using diffusive and convective modes. However, we only had the dialyzer Pecopen 140, a low-flux dialyzer (clears less than 10 kDa), and we did not have the facility of MCO or high-flux dialyzers.

Coupled plasma filtration and adsorption (CPFA) incorporated into the CRRT circuit also has the therapeutic benefit of removing myoglobin. Mario Pezzi *et al.* reported a significant reduction in CPK and myoglobin with improvement in renal functions in four patients with post-traumatic rhabdomyolysis when CPFA was combined with CVVH. However, the rate of decline of blood CPK and Mb levels was slower with CVVH alone compared with CPFA [12].

CytoSorb® is a polymer filter that can adsorb higher-molecular-weight components in plasma. It was mainly studied in the setting of sepsis and septic shock as a cytokine remover. There are limited data on the use of CytoSorb® in the presence of rhabdomyolysis. Olcay Dilken and colleagues reported that myoglobin was efficiently removed using CytoSorb® following exchange with the conventional high-cutoff filter (EMiC-2) in CVVHD, where the high-cutoff filter alone caused therapy failure [13]. Hannah C. Daum and colleagues argued that removing CPK by CytoSorb® is questionable, and the end therapeutic value of removing CK during rhabdomyolysis is unclear. Further, they stated that there is a potential to remove unknown mediators due to muscle damage by CytoSorb® [14].

However, in our patient, we observed a rapid reduction in CPK and myoglobin over 24–48 hours (Fig. 1), which was maintained at low levels after therapy, enabling us to convert to intermittent hemodialysis for renal support and discharge to the ward from the intensive care unit.

## Conclusion

We report a rare manifestation of dengue fever in the form of severe rhabdomyolysis (CPK > 2 million U/L), the highest-ever value reported. In our case, the low-cutoff membranes would have led to poor removal of Mb. Removal of high-molecular-weight proteins such as Mb requires high-volume hemodiafiltration techniques and high-cutoff membranes, and CytoSorb® has a potential benefit by rapidly reducing CPK and myoglobin.

## Data Availability

Not applicable.

## Abbreviations

CPK: Creatine phosphokinase
CVVHDF: Continuous venovenous hemodiafiltration
CRRT: Continuous renal replacement therapy
NS1: Dengue nonstructural protein 1
AKI: Acute kidney injury
LDH: Lactate dehydrogenase
AST: Aspartate transaminase
WCC: White cell count
HB: Hemoglobin
Plt: Platelets
Cr: Creatinine
K: Potassium
ALT: Alanine transaminase
Mb: Myoglobin
CRP: C-reactive protein
EMG: Electromyogram
fT4: Free thyroxine
TSH: Thyroid-stimulating hormone
HIV: Human immunodeficiency virus
CMV: Cytomegalovirus
EBV: Epstein–Barr virus
HSV: Herpes simplex virus
PCR: Polymerase chain reaction
TNFα: Tumor necrosis factor α
IFNα: Interferon-alpha
EBP: Extracorporeal blood purification
CVVH: Continuous venovenous hemofiltration
MCO: Middle cutoff
CPFA: Coupled plasma filtration and adsorption
